# Associations between fear of COVID-19, dental anxiety, and psychological distress among Iranian adolescents

**DOI:** 10.1038/s41405-022-00112-w

**Published:** 2022-06-27

**Authors:** Maryam Tofangchiha, Chung-Ying Lin, Janneke F. M. Scheerman, Anders Broström, Hanna Ahonen, Mark D. Griffiths, Santosh Kumar Tadakamadla, Amir H. Pakpour

**Affiliations:** 1 Social Determinants of Health Research Center, Research Institute for Prevention of Non-Communicable Diseases, Qazvin University of Medical Sciences, Qazvin, Iran; 2 Institute of Allied Health Sciences, Departments of Occupational Therapy and Public Health, and Biostatistics Consulting Center, National Cheng Kung University Hospital, College of Medicine, National Cheng Kung University, Tainan, Taiwan; 3 Department Oral Hygiene, Inholland University of Applied Sciences, Cluster Health, Sport and Welfare, Amsterdam, The Netherlands; 4 Department of Nursing, School of Health and Welfare, Jönköping University, Jönköping, Sweden; 5 Department of Natural Science and Biomedicine, Centre for Oral Health, School of Health and Welfare, Jönköping University, Jönköping, Sweden; 6 International Gaming Research Unit, Psychology Department, Nottingham Trent University, Nottingham, UK; 7 Discipline of Dentistry, Department of Rural Clinical Sciences, La Trobe Rural Health School, La Trobe University, Bendigo, Australia; 8 Violet Vines Marshman Centre for Rural Health Research, La Trobe Rural Health School, La Trobe University, Bendigo, Australia

**Keywords:** Dental anxiety and phobia, Health care

## Abstract

**Objectives:**

The present study evaluated the association of fear of COVID-19 with dental anxiety, oral health-related quality of life (OHRQoL), and psychological distress (depression, anxiety and stress), as well as exploring the mediating role of dental anxiety in the association of fear of COVID-19 with OHRQoL and psychological distress.

**Methods:**

A cross-sectional study was conducted among adolescents in high schools of Qazvin city (Iran) from March-June 2021, recruited through a two-stage cluster sampling method. All the adolescents completed a self-administered survey assessing (i) fear of COVID-19, (ii) depression, anxiety and stress, (iii) OHRQoL, and (iv) dental anxiety. Structural equation modelling was used to evaluate all the hypothesised associations, and the model fit was estimated.

**Results:**

A total of 2429 adolescents participated in the study. The conceptual model fitted the data well. Fear of COVID-19 had a direct effect on dental anxiety (B = 0.316; bias-corrected bootstrapping 95% CI = 0.282, 0.349), depression (B = 0.302; bias-corrected bootstrapping 95% CI = 0.259, 0.347), anxiety (B = 0.289; bias-corrected bootstrapping 95% CI = 0.246, 0.334), stress (B = 0.282; bias-corrected bootstrapping 95% CI = 0.237, 0.328), and OHRQoL (B = −0.354; bias-corrected bootstrapping 95% CI = −0.530, −0.183). Also, dental anxiety mediated the association of fear of COVID-19 with depression, anxiety stress, and OHRQoL.

**Conclusions:**

High levels of fear of COVID-19 were associated with high levels of dental anxiety and poorer OHRQoL. Moreover, fear of COVID-19 was positively associated with anxiety, depression and stress. Increased levels of dental anxiety were also associated with increased anxiety, stress, depression, and poorer OHRQoL.

## Introduction

The most recent data from the World Health Organization indicates that the global burden of COVID-19 cases is still an issue of concern, with over 519 million cases as of mid-May 2022. Approximately 4 million cases were reported every week in August 2021, with the case numbers increasing every week. While the COVID-19 incidence in some countries is on a downward trend, the case numbers have been rising in the Eastern Mediterranean region [1]. In particular, Iran (where the present study was conducted) is among those countries with very high rates of morbidity and mortality associated with COVID-19 [2]. High levels of morbidity and mortality associated with COVID-19 and the uncertainty about new waves with the mutated strains makes it highly likely that many individuals in the general population are experiencing psychological distress. Lockdowns leading to social restrictions and economic recession can also lead to mental stress [3, 4].

A recent systematic review observed high levels of fear due to COVID-19 worldwide, with some disparities observed between the participants genders, regions, and occupations [5]. Such public fear has also been noted during past pandemics [6]. Although fear of COVID-19 could facilitate compliance with the social isolation guidelines, it might also have deleterious impacts if the level of fear is disproportionate to the threat [5]. One such effect of fear is avoidance of care. For instance, 41% of the US adults were estimated to have avoided medical care until mid-2020 due to fear of COVID-19, which also included emergency care [7]. Moreover, anxiety and fear related to COVID-19 could also lead to dental care avoidance [8, 9]. However, there are few studies exploring the effect of fear of COVID-19 on oral health outcomes among adolescents. Moreover, no previous studies have investigated the role of anxiety related to dental treatment in the association between fear of COVID-19 and OHRQoL.

**Fig. 1:**
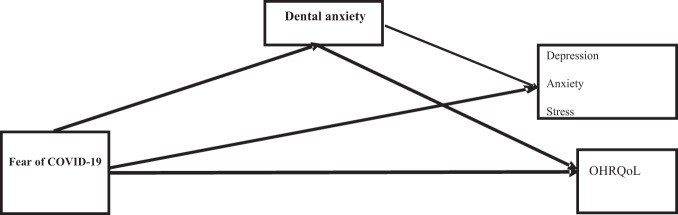
The hypothesised model for the present study. Hypothesised model of the association of fear of COVID-19 and oral health related quality of life (QHRQoL) and psychological distress (including depression, anxiety, and stress) with mediating role of dental anxiety.

Based on the existing empirical research, a theoretical model was hypothesised with plausible pathways examining the associations between fear of COVID-19, psychological distress, dental anxiety, and OHRQoL. Supported by the literature which has demonstrated a strong association between fear of COVID-19 with anxiety and depression [10–13], fear of COVID-19 was assumed to influence psychological distress (depression, anxiety, and stress). Fear of COVID-19 was also hypothesised to influence OHRQoL because poor oral health due to avoidance of care could significantly impact the quality of life (QoL) [14–16]. Dental anxiety is associated with general anxiety levels and depression; therefore, dental anxiety was expected to influence psychological distress [17]. The association between dental anxiety and OHRQoL has been reported in past research [18, 19]. It is also theorised that dental anxiety (i.e., the nervousness associated with receiving dental care [20]), could mediate the association between fear of COVID-19 and OHRQoL. This is because emotional and psychological COVID-19-related stressors [21] as well as uncertainty of oral health care during the COVID-19 pandemic [22] could exacerbate anxiety among dentally anxious individuals. In Iran, dental clinics were closed during the lockdowns, and when the clinics were open, most dentists only considered providing emergency care [23]. The conceptual model is presented in Fig. 1.

The present study evaluated the direct effect of fear of COVID-19 on dental anxiety, OHRQoL, and psychological distress. In addition, the study assessed the mediating role of dental anxiety in the association of fear of COVID-19 with psychological distress and OHRQoL among a sample of Iranian adolescents.

## Methods

A cross-sectional study was conducted among adolescents in high schools in Qazvin, Iran during September to October 2021. The ethics committee of Qazvin University of Medical Sciences approved the study procedure (IR.QUMS.REC.1400.158). Before conducting the study, permission was obtained from the organization for Education at Qazvin and respective schools. Both parents and their adolescents were requested to read the information sheet and sign an online consent form.

### Participants and procedure

A two-stage cluster sampling method was used to recruit participants in the study. Six high schools were randomly selected from the list of all high schools in Qazvin (provided by the organization for Education at Qazvin). At the second stage, all adolescents in the selected schools were invited to participate in the study. All invitations and recruitments were conducted online. Each school has a specific online channel for online learning and teaching in Iran (application of social network software: SHAD). SHAD was used to recruit the study participants.

### Measures

#### Fear of COVID-19 Scale (FCV-19S)

The FCV-19S contains seven items that assess an individuals fear of COVID-19 using a five-point Likert scale (1 = strongly disagree; 5=strongly agree). Therefore, the score range of the FCV-19S is between 7 and 35, where a higher score indicates greater levels of fear of COVID-19 [11]. The psychometric properties of the FCV-19S have been well established for Iranians. For example, the internal consistency of Cronbachs α (α) was 0.82 in a prior Iranian study [24].

#### Depression, Anxiety, Stress Scale-21 (DASS-21)

The DASS-21 contains 21 items that assess three types of psychological distress (depression, anxiety, and stress) among individuals using a four-point Likert scale (0 = did not apply to me at all; 3=applied to me very much or most of the time). Each type of psychological distress has seven items. Therefore, the score range of each psychological distress in the DASS-21 is between 0 and 21, where a higher score indicates greater levels of psychological distress [25]. Internal consistency reliabilities of all three DASS-21 subscales were found to be greater than the required 0.80 for the Persian adaptation of DASS-21 in a previous study (overall DASS-21 scale, α = 0.94; depression subscale, α = 0.85; anxiety subscale, α = 0.85; and stress subscale, α = 0.87) [26].

#### PedsQL Oral Health Scale

The PedsQL Oral Health Scale contains five items that assess an individuals OHRQoL using a five-point Likert scale (0 = never a problem; 4=almost always a problem). The five-point Likert scale is then linearly transformed into a 0–100 scale. The score range of the PedsQL Oral Health Scale is between 0 and 100, where a higher score indicates better OHRQoL [27]. The psychometric properties of the PedsQL Oral Health Scale have been well established for Iranians. For example, the Cronbachs α was 0.79 in a prior Iranian study among children and adolescents [28].

#### Modified Dental Anxiety Scale (MDAS)

The MDAS contains five items that assess an individual's dental anxiety using a five-point Likert scale (1 = not anxious; 5=extremely anxious) with minimum and maximum possible scores of 5 and 25 respectively. A higher score indicates greater levels of dental anxiety [29]. The Persian version of MDAS has been found to be reliable (α = 0.80) among adolescents [30].

### Data analysis

Socio-demographic characteristics, and mental health outcomes were firstly analysed using descriptive statistics. Then, Pearson correlation coefficients were calculated to understand the associations between the studied variables. Finally, structural equation modelling (SEM) was performed using the full information maximum likelihood (FIML) estimation with bias-corrected confidence intervals (CI) to examine if the data fit the proposed model well. In the SEM, 5000 bootstrapping resamples were used, and the fit of the proposed model was assessed using several indices. More specifically, non-significant χ^2^ test indicates a satisfactory fit. However, given that χ^2^ test is sensitive to a large sample size (e.g., over 2000 participants in the present study), the following indices with cutoffs were also used. That is, comparative fit index (CFI) and Tucker Lewis index (TLI) larger than 0.9 together with standardised root mean square residual (SRMR) and root mean square error of approximation (RMSEA) less than 0.08 indicate satisfactory fit. In the SEM, the significance of any direct or indirect path was supported when the bias-corrected bootstrapping 95% CI does not include 0. The SEM was conducted using the IBM AMOS version 24 (IBM SPSS. Inc., Chicago, IL) and the rest of the analyses were conducted using the IBM SPSS version 24 (IBM Corp., Armonk, NY). A biostatistician was consulted for the data analysis.

## Results

Among the 2429 participants (mean [SD] age=15.28 [2.79] years), less than half were males (*n* = 1068; 43.97%). Moreover, less than one-third of the participants had visited a dentist in the past year (*n* = 800; 32.9%), and just over one-fifth of the participants had never visited a dentist (*n* = 509; 21.0%). Regarding their oral hygiene behaviour, less than a quarter of the participants brushed their teeth twice per day (*n* = 507; 21.0%), and less than one-fifth used dental floss once per day (*n* = 397; 16.3%). Moreover, the participants' mean scores on different mental health outcomes were 17.52 (SD = 6.05) for dental anxiety; 73.44 (SD = 27.54) for OHRQoL; 22.96 (SD = 6.96) for fear of COVID-19; 9.65 (SD = 3.93) for depression; 9.17 (SD = 4.98) for anxiety; and 8.84 (SD = 5.03) for stress (Table 1).

**Table 1:** Participants characteristics (N = 2429).

	n (%) or Mean ± SD
Demographics	
Age	15.28 ± 2.79
Gender (male)	1068 (43.97%)
Number of family member	
≤4	1676 (69.0%)
5–7	482 (19.84%)
>7	271 (11.16%)
Fathers' educational year	10.22 ± 5.30
Mothers' educational year	8.23 ± 5.37
Last time visit dentist	
<6 months	296 (12.2%)
6 months to 1 year	504 (20.7%)
1–2 years	679 (28.0%)
>2 years	441 (18.2%)
Never	509 (21.0%)
Frequency of using dental brush	
Never	181 (7.5%)
Less than once per month	124 (5.1%)
Less than once per week	131 (5.4%)
Once per week	358 (14.1%)
Once per day	1128 (46.9%)
Twice per day	507 (21.0%)
Frequency of using dental floss	
Never	984 (40.5%)
Less than once per month	437 (18.0%)
Less than once per week	261 (10.7%)
Once per week	350 (14.4%)
Once per day	397 (16.3%)
Health outcomes	
Dental anxiety^a^	17.52 ± 6.04
Oral health-related quality of life^b^	73.44 ± 27.54
Fear of COVID-19^c^	22.96 ± 6.96
Depression^d^	9.65 ± 3.93
Anxiety^d^	9.17 ± 4.98
Stress^d^	8.84 ± 5.03

^a^Assessed using Modified Dental Anxiety Scale.

^b^Assessed using PedsQL Oral Health Scale.

^c^Assessed using Fear of COVID-19 Scale.

^d^Assessed using Depression, Anxiety, Stress Scale-21.

The correlations between the studied variables were all significant (all *p* values <0.01). More specifically, dental anxiety, fear of COVID-19, depression, anxiety, and stress were positively associated with each other with moderate to large effect sizes (*r* = 0.294–0.797). OHRQoL was negatively associated with dental anxiety, fear of COVID-19, depression, anxiety, and stress with small to moderate effect sizes (*r* = −0.146 to −236) (Table 2).

**Table 2:** Correlation matrix among tested variables.

				*r*		
	1.	2.	3.	4.	5.	6.
1. Dental anxiety^a^	—	0.362^**^	0.294^**^	0.287^**^	0.297^**^	−0.186^**^
2. Fear of COVID-19^b^		—	0.374^**^	0.357^**^	0.350^**^	−0.146^**^
3. Depression^c^			—	0.797^**^	0.609^**^	−0.234^**^
4. Anxiety^c^				—	0.574^**^	−0.229^**^
5. Stress^c^					—	−0.236^**^
6. OHRQoL^d^						—

^a^Assessed using Modified Dental Anxiety Scale.

^b^Assessed using Fear of COVID-19 Scale.

^c^Assessed using Depression Anxiety Stress Scales.

^d^Assessed using PedsQL Oral Health Scale.

^**^p < 0.05.

The proposed model was supported by the satisfactory fit indices in the SEM (CFI = 0.999, TLI = 0.986, SRMR = 0.007, RMSEA = 0.031), except for the significant χ^2^ test (χ^2^ [*df*]= 10.201 [3], *p*-value of χ^2^ test=0.02). Moreover, significant direct effects were found in the associations between fear of COVID-19 and the following variables: dental anxiety (B = 0.316; bias-corrected bootstrapping 95% CI = 0.282, 0.349), depression (B = 0.302; bias-corrected bootstrapping 95% CI = 0.259, 0.347), anxiety (B = 0.289; bias-corrected bootstrapping 95% CI = 0.246, 0.334), stress (B = 0.282; bias-corrected bootstrapping 95% CI = 0.237, 0.328), and OHRQoL (B = −0.354; bias-corrected bootstrapping 95% CI = −0.530, −0.183). Significant direct effects were also found in the associations between dental anxiety and the following variables: depression (B = 0.220; bias-corrected bootstrapping 95% CI = 0.174, 0.266), anxiety (B = 0.217; bias-corrected bootstrapping 95% CI = 0.183, 0.276), stress (B = 0.230; bias-corrected bootstrapping 95% CI = 0.183, 0.276), and OHRQoL (B = −0.697; bias-corrected bootstrapping 95% CI = −0.892, −0.519). Additionally, dental anxiety was found to be a significant mediator in the associations between fear of COVID-19 and the following variables: anxiety, depression, stress, and OHRQoL (Table 3).

**Table 3:** Predictors for both oral-health-related Quality of Life (OHRQoL) and psychological distress.

Predictors	B	Bootstrap SE	Bias-corrected 95% CI (lower bound)	Bias-corrected 95% CI (upper bound)
Direct effects				
Fear of COVID-19 on dental anxiety	0.316	0.017	0.282	0.349
Fear of COVID-19 on depression	0.302	0.023	0.259	0.347
Fear of COVID-19 on anxiety	0.289	0.023	0.246	0.334
Fear of COVID-19 on stress	0.282	0.023	0.237	0.328
Fear of COVID-19 on OHRQoL	−0.354	0.089	−0.530	−0.183
Dental anxiety on depression	0.220	0.024	0.174	0.266
Dental anxiety on anxiety	0.217	0.023	0.174	0.264
Dental anxiety on stress	0.230	0.024	0.183	0.276
Dental anxiety on OHRQoL	−0.697	0.095	−0.892	−0.519
Indirect effects of fear of COVID-19 through dental anxiety				
Anxiety	0.069	0.009	0.053	0.088
Depression	0.070	0.009	0.053	0.086
Stress	0.073	0.009	0.057	0.092
OHRQoL	−0.220	0.033	−0.290	−0.159

*B* unstandardized coefficient, *SE* standard error, *CI* confidence interval.

## Discussion

The present study explored the association of fear of COVID-19 with psychological distress and OHRQoL along with exploring the mediating role of dental anxiety in this association. It was found that fear of COVID-19 was significantly associated with psychological stressors and OHRQoL. In addition to the direct effect, fear of COVID-19 also had an indirect effect on OHRQoL through dental anxiety.

To the best of the present authors’ knowledge, this is the first study to evaluate the role of fear of COVID-19 on oral health outcomes using a standardised instrument (i.e., the FCV-19S). As hypothesised, fear of COVID-19 was found to have direct effect on all the three psychological stressors (anxiety, depression, and stress) along with dental anxiety and OHRQoL. The impact of fear of COVID-19 on anxiety, depression and stress has widely been tested and is now evident in the extant literature [31]. Studies have also found the impact of COVID-19 on self-harm and suicidal ideation throughout the developing world [32, 33]. Coping mechanisms such as mindfulness [31], optimism, and resilience [34] could help reduce the impact of fear of COVID-19 on psychological distress. Unsurprisingly, the present study found increased fear of COVID-19 to be associated with higher levels of dental anxiety among Iranian adolescents.

Although previous personal traumatic experiences or indirect conditioning from other sources are important causes of dental anxiety, research indicates that neuroticism and extraversion are the most important personality traits that are related to dental anxiety [35]. While extraversion is associated with excitement seeking, neuroticism is associated with anxiety, hostility, and depressive symptoms [35]. Experimental research has also demonstrated that neurotic individuals quickly acquire fear, and that these individuals also find difficulty learning when the threat to a stimulus is unpredictable, which very well applies to the current COVID-19 situation [36, 37]. In addition to the unpredictability of COVID-19 situation, the fear of transmission during dental treatment could further exacerbate dental anxiety.

It was postulated that the direct effect of the fear of COVID-19 on OHRQoL could potentially be mediated through lack of treatment or avoidance of care, which was unfortunately not assessed in the present study. Longitudinal studies evaluating the impact of dental care avoidance on OHRQoL in adolescents is lacking. Nevertheless, a large study involving Swedish adults observed that avoidance of care, albeit due to cost, caused impaired OHRQoL [38].

Dental anxiety served as a mediator in the path of association of fear of COVID-19 with psychological outcomes and OHRQoL. The effect of dental anxiety on OHRQoL among children and adolescents is apparent from the existing evidence [39] but it was interesting to note the positive association of dental anxiety with depression, anxiety, and stress. This may be explained by three potential mechanisms. Firstly, dental anxiety could influence self-perceived vitality and general wellbeing which in turn are negatively associated with depression, anxiety, and stress [40]. On the other hand, high levels of dental anxiety coupled with confusion around the process of seeking dental care during the current pandemic could have led to psychological distress. Lastly, those individuals with high levels of dental anxiety tend to show high levels of neuroticism, which is accompanied by anxiety and depression [35]. A survey among dental professionals in Iran found that most Iranian dentists limited their work hours and scope of practice for emergency procedures during the COVID-19 pandemic [23]. They also reported an increased demand for remote consultations [23]. Consequently, there is an urgent need to reorganise the dental care delivery system with a focus on telehealth services to reduce the burden of oral disease and to promote their overall wellbeing.

The strengths of the present study include its large sample of Iranian adolescents recruited using a probability sampling technique; thereby, the findings of the present study have high external validity. Moreover, all the measures used in the present study were previously validated for use in Iranian populations. Despite these strengths, there are some limitations that should be mentioned. Firstly, no causal relationships can be determined for the tested associations due to the cross-sectional nature of the study. Secondly, objective measures of oral disease burden or treatment history could not be included in the conceptual framework due to the lack of opportunity to collect such data. Future studies should attempt to explore the complex relationships between the fear of COVID-19 with subjective and objective measures of oral health outcomes embedded within a comprehensive conceptual framework. Also, the cause-and-effect relationship of the associations observed in the present study need to be determined. Finally, neither the FCV-19S nor DASS-21 have been specifically validated for use among adolescent samples, although their psychometric properties were found to be good among adolescents.

In conclusion, high levels of fear of COVID-19 were associated with high levels of dental anxiety and poorer OHRQoL. In addition, fear of COVID-19 was positively associated with anxiety, depression, and stress. Increased levels of dental anxiety were also associated with increased anxiety, stress, depression, and poorer OHRQoL. Dental anxiety was also found to play a mediating role in the association between fear of COVID-19 and OHRQoL.
